# Toddlers and terriers: a One Health partnership to combat antimicrobial resistance

**DOI:** 10.1111/jsap.70136

**Published:** 2026-04-12

**Authors:** M. Prodanuk, F. Emdin

**Affiliations:** ^1^ Centre for Global Child Health The Hospital for Sick Children Toronto Ontario Canada; ^2^ Division of Infectious Diseases, Department of Paediatrics, Temerty Faculty of Medicine The Hospital for Sick Children, University of Toronto Toronto Ontario Canada; ^3^ Institute of Health Policy, Management and Evaluation, Dalla Lana School of Public Health University of Toronto Toronto Ontario Canada; ^4^ Global Strategy Lab York University Toronto Ontario Canada

“Do you want to provide a free consult?” I ask Michael as I slide into the wobbly metal chair and kick my flattened gym bag under the table. The pita shop is crowded as always, and the table rocks slightly every time either of us touches it.

He raises an eyebrow. “Depends. Do I get paid in garlic sauce?”

“That's above my pay grade”, I say, pushing damp hair out of my face.

Our weekly post‐workout pita lunch has become our ritual over the past year. We met in graduate school and are both studying clinical epidemiology. I'm a shelter medicine veterinarian patching up neglected dogs and anxious cats and Michael is a paediatric infectious diseases physician. Despite different clinical backgrounds, our “One Health” friendship blossomed over a shared interest in antimicrobial resistance (AMR) in humans and animals. As it turned out, Michael's patients were struggling with the same antibiotic‐resistant “super bugs” as my furry friends.

“Of course”, he tells me, grinning across the table. He cracks his pop with a hiss of escaping air, “What's up in the world of puppies and kittens?”

“I have a middle‐aged terrier mix with its third urinary tract infection (UTI) in three months. The owners finally agreed to a urine culture”.

I scroll through my phone until I find the results and rotate the screen towards him. “It grew *E. coli*, resistant to most antibiotics; looks like an extended‐spectrum beta‐lactamase (ESBL)‐producer”. I tell him, “And before you ask”, I cut him off with a gentle wave of the hand, “No obvious underlying reason for the frequent UTIs. No stones on ultrasound, diabetes or other risk factors”. I continue, “I haven't seen this dog before, but her regular vet prescribed cephalexin for 14 days a couple of times. Sadly, resistant to that now too, so I'm going to prescribe a third‐generation cephalosporin called cefovecin”.

“I haven't heard of that antibiotic before”, Michael quips, “You vets get all the new fancy drugs! Those previous 14‐day courses sound long, though, and I worry they may have contributed to resistance”.

“Agreed”, I continue, “and that's why I'm debating the optimal duration. Since this is a recurrent case, should I stick with the standard three to five days (Weese et al., 2019)? Or go longer?”

Michael ponders, “Well, I'm no expert in terriers, but in teens and adults, three days is often enough for uncomplicated UTIs (Nelson et al., 2024), even when they've had UTIs before. We usually reserve longer durations for younger children and infections that have ascended to the kidneys”.

He pauses, scratching his head: “You know, your terrier reminds me of this little girl, a toddler, that I follow in clinic for frequent UTIs. Her doctor was prescribing longer and longer courses of antibiotics, but the infections kept coming back. She even had to be hospitalised for intravenous antibiotics once, since the bacteria were resistant to all oral options. I think ESBL *E. coli* grew in her urine too…”.

Overcome with excitement, I interrupt Michael mid‐sentence. “You know what is weird? The owners of my terrier said their three‐year‐old daughter also has recurrent UTIs. I don't know much about human diseases, but I have seen reports of resistant skin organisms transferring between owners and their dogs, including methicillin‐resistant *Staphylococcus aureus* (MRSA) (Manian, 2003). I've also heard of resistant UTI‐causing gram‐negatives passing between humans and their pets, some of which can withstand even the most broad‐spectrum antibiotics (Menezes et al., 2024). I pause, looking down at the culture report on my phone. I'm not saying that's what's happening here, but you have to wonder…”.

He doesn't get a chance to answer because the kitchen bell dings and someone shouts our order number. I weave through crowded tables to grab our pitas, doing my best not to drop them or wipe out on the tile floor. As I slide back into my seat, Michael leans over our meal, “In people, we will often consider specific infections after animal contact, such as rabies from a bat bite or bartonellosis from a cat scratch, but we don't often consider the risk from organisms in the animal's microbiome that may have become antibiotic resistant”.

I chime in, “If these bacteria go back and forth between animal and owner, treatment could get really challenging”. Sighing, I add, “Treat one just to be re‐infected by other”.

“Exactly!” he exclaims with a mouth half‐full of food, “For my patients with recurrent infections from these resistant bugs, it could be helpful to screen other household members, maybe even the pets. We sometimes do that for the families of children with repeated MRSA infections”.

Finished chewing, Michael adds with a concerned face, “I'm worried about these carbapenem‐resistant bacteria that are emerging too, since our broadest spectrum antibiotics don't touch them (Tamma et al., 2024). We have started seeing children hospitalised with severe infections from these bugs, and the outcomes aren't always positive. Sure, there are a few new antibiotics available to treat them, but sadly in Canada, many are not approved, so therapy is often delayed as we request special access. And it's possible a child could pass these bugs to their pet and vice versa”.

I nod along as Michael speaks. Anecdotally, I've wondered about rising resistance in my own practice too, and some studies from the US (Cooke et al., 2002) and Canada (Prescott et al., 2002) confirm similar trends in dogs and cats. I can't help but wonder how much of this is linked to the use of antibiotics that aren't always first‐line choices in veterinary medicine. For example, third‐generation cephalosporins, a critically important antibiotic class in human medicine, are frequently prescribed without justification in cats and dogs (Burke et al., 2017), which may be driving resistance.

Now wiping his hands, Michael continues, “We need veterinarians, physicians, public health officials, and others to team up to improve antimicrobial use and sort out these cases of animal‐human transmission. Otherwise, how can we break the vicious cycle? Without integrated human‐animal AMR surveillance, cases of owner‐to‐pet transmission of resistant bacteria may be difficult to catch. We need real‐time ‘One Health’ reporting, so public health responses can match. You and I both know there have been some success stories with this approach (Delpy et al., 2024); it can be done”.

“Totally agree”, I say as we stand and toss our napkins into the garbage, gym bags slung over tired shoulders. We push open the door and walk back onto the busy downtown Toronto sidewalk.

Over the din of traffic, Michael continues, “We should get some of our colleagues in vet med, infectious diseases, microbiology, and public health together – see what we can do locally. Same place, same time next week to hash out a plan?”

“You're on!” I exclaim, giving him a too‐hard pat on the back.

While we have improved the integration of AMR data in some contexts (Public Health Agency of Canada, 2022), most infrastructure for tracking AMR organisms is still siloed. Human, veterinary and environmental labs are often separate and don't report data together except at a high level and retrospectively. While resistant bacteria may be jumping between animals and humans, without improving surveillance, providers may be left in the dark while patients struggle with difficult‐to‐treat infections. Similarly, while antimicrobial stewardship guidelines exist to help clinicians select appropriate antibiotics and treatment durations, without integrated systems to stay on top of emerging resistance patterns, these guidelines may lag behind. If our data systems were integrated, real‐time reporting of resistant organisms could improve and help inform actionable antimicrobial stewardship, one infection at a time.

Michael and I part ways at the corner. He heads west, and I head east, both of us returning to our separate clinics, our separate patients and our separate problems. Except they aren't separate at all, and until our systems reflect an integrated “One Health” approach to combat AMR, we'll stay one step behind the bacteria, reactive instead of proactive and running back and forth between toddlers and terriers.

## Author contributions

**M. Prodanuk:** Conceptualization; methodology; writing – original draft; writing – review and editing. **F. Emdin:** Conceptualization; methodology; writing – original draft; writing – review and editing.

## Conflict of interest

The authors declare that they have no competing interests.

## Funding

Not funded.

## Data Availability

Data sharing not applicable to this article as no datasets were generated or analysed during the current study.
